# RET Signaling Pathway in Human Cancer: Oncogenic Mechanisms, Selective Inhibitors, and Emerging Resistance Strategies

**DOI:** 10.3390/ijms27073180

**Published:** 2026-03-31

**Authors:** Spencer Streit, Aala Dweik, Amen Mahtab, Sundas Ali, Abat Khan, Matthew Salzberg

**Affiliations:** Hematology/Oncology Department, Memorial Healthcare System, Pembroke Pines, FL 33028, USA; sstreit@mhs.net (S.S.); adweik@mhs.net (A.D.); amahtab2022@fau.edu (A.M.); sunali@mhs.net (S.A.); abkhan@mhs.net (A.K.)

**Keywords:** RET, tyrosine kinase, targeted therapy, resistance mechanisms

## Abstract

The proto-oncogene *Rearranged During Transfection* (RET) encodes a receptor tyrosine kinase that is essential for neural, renal, and thyroid development. Pathogenic RET alterations, including mutations and fusions, drive oncogenesis, most notably medullary and papillary thyroid carcinomas and non-small cell lung cancer, by constitutively activating downstream RAS–MAPK, PI3K–AKT, and JAK–STAT signaling. Early multi-kinase inhibitors such as vandetanib and cabozantinib demonstrated modest efficacy with significant toxicity, whereas the selective RET inhibitors selpercatinib and pralsetinib have achieved improved response rates and tolerability. However, resistance remains a key clinical challenge, arising from secondary RET mutations and bypass signaling via MET or EGFR pathways. Continued investigation into next-generation inhibitors and rational combination therapies aims to overcome resistance and optimize treatment sequencing, advancing precision oncology for RET-altered malignancies. Nonetheless, resistance, driven by secondary mutations and bypass signaling, presents a major therapeutic challenge. Ongoing development of next-generation inhibitors and combination strategies aims to overcome resistance and improve patient outcomes.

## 1. Introduction

The *RET* proto-oncogene encodes a receptor tyrosine kinase (RTK) that is essential for embryonic development, neuronal differentiation, and tissue homeostasis, especially in the nervous system, kidneys, and thyroid gland. The RET receptor is activated by glial cell line-derived neurotrophic factor (GDNF) family ligands (GFLs), which interact with glycosylphosphatidylinositol (GPI)-anchored co-receptors (GFRα1–4) [1]. RET dimerization and autophosphorylation facilitate interaction, activating downstream pathways such as RAS-MAPK, PI3K-AKT, and JAK-STAT, which regulate cell proliferation, survival, and differentiation.

Despite its essential physiological functions, dysregulation of RET signaling due to genetic mutations, gene fusions, or abnormal expression has been implicated in various human malignancies. *RET* alterations are most commonly linked to thyroid cancers, including papillary thyroid carcinoma (PTC) and medullary thyroid carcinoma (MTC), as well as non-small cell lung cancer (NSCLC) [2]. *RET*/*PTC* gene fusions in thyroid cancers are often detected in radiation-induced PTCs. In contrast, germline RET mutations serve as the primary oncogenic drivers in multiple endocrine neoplasia type 2 (MEN2), an inherited cancer syndrome that predisposes individuals to MTC, pheochromocytomas, and parathyroid hyperplasia. Additionally, somatic *RET* mutations contribute to sporadic cases of MTC and other solid tumors.

*RET* oncogenic activation primarily occurs through three distinct mechanisms: (1) RET fusions (such as *RET*/*PTC* in thyroid cancer and *RET* fusions in NSCLC), (2) point mutations in *RET* (commonly observed in MEN2-associated medullary thyroid carcinoma), and (3) *RET* overexpression in epithelial malignancies, including breast and pancreatic cancer. *RET* fusions lead to constitutive kinase activation by removing regulatory elements and promoting dimerization, resulting in ligand-independent RET activation. Similarly, *RET* point mutations—especially those in the intracellular kinase domain—cause constitutive autophosphorylation, further enhancing its oncogenic signaling capabilities.

RET activation significantly influences tumorigenesis and cancer progression by promoting cell survival, proliferation, and resistance to apoptosis. The downstream MAPK/ERK and PI3K/AKT pathways associated with RET affect tumor growth, metastasis, and therapeutic resistance. Furthermore, abnormal RET activation is linked to immune evasion and the tumor microenvironment through the modulation of inflammatory cytokines and immune checkpoint molecules, highlighting its broader implications beyond mere cancer cell proliferation [3].

Given its oncogenic potential, RET has emerged as a therapeutic target in multiple cancers. Early treatment strategies involved multi-kinase inhibitors (MKIs) such as vandetanib and cabozantinib, which were initially approved for advanced medullary thyroid cancers [4]. While these MKIs demonstrated clinical efficacy, their off-target toxicities and limited selectivity diminished their therapeutic benefits.

Recent advances in precision oncology have led to the development of highly selective RET inhibitors, such as selpercatinib (LOXO-292) and pralsetinib (BLU-667), which received FDA approval in 2020 for RET-driven NSCLC and thyroid cancers [5]. These next-generation inhibitors exhibit superior selectivity, potency, and reduced toxicity compared to older MKIs, significantly enhancing the progression-free survival (PFS) and overall response rates (ORR) in RET-driven cancers [6].

Despite these advances, resistance to RET inhibitors remains a significant challenge. Mechanisms of acquired resistance include secondary *RET* mutations, activation of bypass signaling pathways, and histological transformation. For example, gatekeeper mutations in *RET*, such as *RET* G810 mutations, have been identified as critical drivers of resistance to selpercatinib and pralsetinib, highlighting the necessity for developing next-generation RET inhibitors. Several novel RET inhibitors, including BOS172738, TPX-0046, and LOX-18228, are currently in clinical trials, offering hope for patients who develop resistance to first-line RET inhibitors [7].

The RET signaling pathway is vital in cancer pathogenesis, particularly in thyroid and lung cancers. Selective RET inhibitors have transformed the treatment landscape for RET-driven malignancies, significantly improving patient outcomes. However, emerging resistance mechanisms pose a considerable challenge, necessitating continuous innovation in RET-targeted therapies. Future research needs to focus on combination strategies that integrate immune checkpoint inhibitors, chemotherapy, and novel small-molecule inhibitors to enhance therapeutic efficacy and delay resistance. Additionally, biomarker-driven patient selection will be essential for optimizing treatment outcomes and personalizing therapies for RET-positive cancers.

This review thoroughly analyzes RET signaling, its oncogenic role, and the evolving landscape of RET-targeted therapies. It also emphasizes the new challenges and opportunities in the fight against RET-driven cancers.

## 2. Role of RET Signaling in Human Cells

### 2.1. RET Proto-Oncogene

The RTK, *RET*, was first identified in 1985 as a transforming gene derived from human lymphoma DNA transfected into NIH3T3 [8]. The resulting fusion of two human DNA sequences formed the oncogenic gene, later named *RET* due to its rearrangement during transfection [9]. *RET* continues to refer to the proto-oncogene encoding this tyrosine kinase [10], located on chromosome 10q11.2 [11].

**Figure 1:**
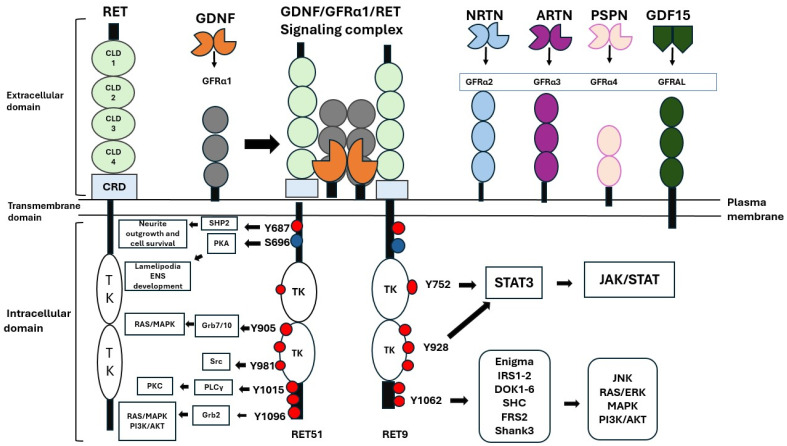
The structure, activation and downstream signaling pathways of receptor RTK RET. Its extracellular region encompasses four cadherin-like domains and a cysteine-rich domain. The protein also includes a single segment that spans the plasma membrane. Internally, RET contains an intracellular domain, which features a large tyrosine kinase (TK) domain embedded within its structure. RET is activated by *GFLs* or *GDNF* family ligands, such as *GDNF*, *NRTN*, *ARTN*, and *PSPN*, which bind to *GFRα1*, *GFRα2*, *GFRα3*, and *GFRα4*, respectively. The *GDNF/GFRα1/*RET signaling complex is crucial for spermatogonial stem cell self-renewal, survival, and development of the kidney, urinary tract, and enteric nervous system. The cytokine *GDF15*, which responds to stress, engages with *GFRAL* and initiates RET activation, playing a role in the regulation of food intake and body mass. RET 51, the longer isoform consisting of 658–1114 amino acids, and RET 9, the shorter isoform comprising 1072 amino acids, exhibit identical sequences for the initial 1063 amino acids at their N-terminal ends. The sole distinction between these two isoforms lies in their C-terminal regions. Red circles indicate sites of tyrosine autophosphorylation, whereas blue circles indicate the location of serine 696. The summary and abbreviations of the adapter molecules linked to each phosphorylation site are given below. Src homology 2 domain containing transforming protein (SHC); fibroblast growth factor receptor substrate 2 (FRS2); insulin receptor substrate 1/2 (IRS1/2); Grb2; phospholipace C γ (PLCγ); Src homology region 2 domain-containing protein tyrosine phosphatase-2 (SHP2); growth factor receptor-bound protein 7 and 10 (Grb7 and Grb10), signal transducer and activator of transcription 3 (STAT3); protein kinase A (PKA); protein kinase C (PkC); STAT; mitogen-activated protein kinase (MAPK); extracellular signal-regulated kinase (ERK), phosphoinositide 3 kinase (PI3K); multiple ankyrin repeat domains 3 (SHANK3); c-Jun N-terminal kinase (JNK); growth differentiation factor-15 (GDF15); GDNF family receptor α-like (GFRAL); GDNF; neurturin (NRTN); artemin (ARTN); persephin (PSPN); CRD; and CLD. Adapted from Mechanisms of Disease: cancer targeting and the impact of oncogenic RET for medullary thyroid carcinoma therapy/RET Proto-Oncogene/Intracellular RET signaling pathways activated by *GDNF*.

The *RET* gene encodes a protein that consists of an extracellular domain, a transmembrane segment, and an intracellular tyrosine kinase domain. The extracellular region includes CLD 1–4 and a CRD containing a key calcium-binding site between CLD2 and CLD3, which is essential for receptor function [12] (Figure 1).

RET undergoes alternative splicing at the 3′ region, producing three isoforms—RET9, RET43, and RET51—with carboxy-terminal tails of 9, 43, and 51 amino acids, respectively [2]. The longest isoform, RET51, comprises 1114 amino acids: an extracellular domain (aa 29–635), a hydrophobic transmembrane region (aa 636–657), and an intracellular kinase domain (aa 657–1114) [13]. The mature 170-kDa protein results from calcium-dependent conversion of the 150-kDa immature RET in the endoplasmic reticulum. RET’s intracellular region includes 18 tyrosine residues—two in the juxtamembrane domain, 11 in the kinase domain, and five in the C-terminal region [13]. *RET* is crucial for the development of the excretory and nervous systems [14]. It is expressed in the ureteric bud, nephric ducts, and enteric neural crest cells during embryogenesis, as well as autonomic and dorsal root ganglia [15]. In adults, *RET* expression persists in neurons of the central and peripheral nervous systems but is absent in renal tissues [15].

### 2.2. Activation and Downstream Signaling

RET activation occurs through GFLs, part of the TGF-β superfamily, which includes *GDNF*, *NRTN*, *ARTN*, and PSPN [16,17]. These ligands first bind to specific GFRα co-receptors (GFRα1–4) anchored to the cell membrane via GPI linkages [18]. Ligand–co-receptor complexes recruit two RET molecules into lipid rafts, promoting dimerization and trans-autophosphorylation of intracellular tyrosine residues [17]. *GDNF–GFRα1/*RET signaling supports kidney development and enteric nervous system formation, while *GDF15–GFRAL–*RET signaling regulates energy balance and appetite [2,17]. Up to 14 of the 18 tyrosines can undergo phosphorylation, recruiting adaptor proteins that activate pathways regulating growth, survival, and differentiation [19].

Among C-terminal residues, Y1015, Y1029, and Y1062 are shared by both RET9 and RET51, while Y1096 and Y1102 are unique to RET51 [20]. Phosphorylated Y1062 is a critical docking site for SHC, FRS2, DOK, and IRS1/2, which activate RAS/ERK and PI3K/AKT signaling pathways involved in cell survival and differentiation [20,21,22]. Y1062 also interacts with Dok1 and Nck to activate JNK signaling [23].

Y752 and Y928 recruit STAT3, enabling its phosphorylation and nuclear translocation for transcriptional activation [3]. Y905 binds Grb7/10, stimulating Ras/MAPK signaling for proliferation [24,25]. Y1096 (unique to RET51) recruits GRB2, activating MAPK and PI3K cascades; its presence compensates for Y1062F loss-of-function mutations [22]. Y1015 engages PLCγ, triggering PKC activation and Ca^2+^ release, which are critical for urinary tract formation; mutations here mimic CAKUT features [20]. Y981 binds Src kinase, driving neuronal survival and neurite outgrowth; mutation (Y981F) disrupts GDNF-mediated responses [26]. S696, phosphorylated by PKA, regulates Rac1 activation and lamellipodia formation, which is essential for enteric neuron migration [27]. Y687 recruits SHP2, activating PI3K/AKT signaling; mutation (Y687F) impairs neuronal survival and extension [28].

Finally, the *GDNF–*RET pathway regulates spermatogonial stem cell (SSC) renewal and spermatogenesis. Sertoli cell-derived GDNF maintains SSC maturation, and RET pathway defects cause impaired sperm production, underscoring the essential role of the GFL–GFRα1–RET axis in male fertility [3].

### 2.3. Mutations/Aberrations

The *RET* gene’s mutations are linked to alterations in essential cellular functions, such as proliferation, invasion, and migratory behavior [29].

RET oncogenic activation arises mainly from gene fusions and point mutations, gain-of-function events that potentiate RET activity across diverse malignancies [12,30].

These genetic alterations can lead to the activation of RET, triggering the onset of various hereditary and non-hereditary disorders [21].

Congenital abnormality Hirschsprung disease (HSCR) is associated with mutations that cause a loss of function in the RET gene [31].

#### 2.3.1. Point Mutations

Activating mutations in *RET*, whether germline or somatic, are prevalent among individuals with MTC [32].

Germline mutations in the *RET* proto-oncogene are linked to MEN2. MEN2 is classified into three subtypes, including MEN2A, FMTC (familial medullary thyroid carcinoma), and MEN2B. Individuals with these genetic variations face a 70% to 100% likelihood of developing medullary thyroid carcinoma by the age of 70 [33].

The mutations associated with multiple endocrine neoplasia type 2 are predominantly found in two key areas of the RET protein: the cysteine-rich extracellular domain and the intracellular TK domains [34].

Mutations affecting the cysteine-rich region of the RET extracellular domain frequently involve the cysteine residues located at positions 609, 611, 618, and 620 in exon 10, as well as positions 630 and 634 in exon 11. These mutations substitute cysteine residues with other amino acids, thereby reducing intramolecular disulfide bond formation. This change promotes the creation of RET homodimers via intermolecular disulfide linkages between RET monomers. As a result, RET undergoes constitutive activation without ligand binding [35].

*RET* mutations within the cysteine-rich domain are the most common in the cases of MEN2A and FMTC. Notably, around 85% of family members affected by MEN2A display missense mutations occurring at codon 634. Furthermore, specific mutations are documented in certain MEN2A and/or FMTC families. These include the alterations Glu768Asp, Leu790Phe, Tyr791Phe, Val804Met/Leu, and Ser891Ala found in exons 13–15 of the RET kinase domain, as well as the G533C mutation in the RET extracellular domains exon 8 [12].

Mutations in *RET* tyrosine kinase domain (TKD), specifically Met918Thr in exon 16 and Ala883Phe in exon 15, trigger a structural shift in the catalytic core. This change enhances ATP binding and activates RET without the need for dimerization. These TKD alterations are more commonly observed in MEN2B, with M918T occurring in 95% of cases and A883F in 2–3% of instances [10]. In 40–70% of sporadic medullary thyroid carcinomas, somatic activating *RET* mutations are identified, which are linked to more aggressive tumor behavior. Among these genetic alterations, the *RET* M918T mutation is the most frequently encountered [32].

*RET* mutations that are activating in nature result in intensified signaling cascades through multiple downstream effectors, particularly the MAPK and PI3K pathways. This enhanced signaling promotes increased cellular proliferation and growth that is not dependent on anchorage [36].

#### 2.3.2. HSCR

Hirschsprung’s disease (HSCR) results from inactivating point mutations or deletions in the RET gene [2].

HSCR is an ENS developmental abnormality marked by the insufficiency of ganglion cells in the distal portion of the intestine, affecting 1 in 5000 births. This condition stems from the unsuccessful migration of neural crest cells. The sooner migration is halted, the more extensive the aganglionic portion becomes. Genetic alterations in the *RET* gene are accountable for 15–20% of sporadic cases and 50% of familial instances of HSCR [37].

Mutations in the *RET* gene can be found across its entire coding sequence and include various types, such as deletions, insertions, frameshifts, non-sense, and missense mutations. Most of these genetic alterations result in either a decrease in RET protein levels or a loss of RET functionality. This suggests that HSCR is likely caused by RET haploinsufficiency [38].

#### 2.3.3. RET Fusion

In the late 1980s, scientists first discovered *RET* alterations when they identified an oncogenic RET fusion in PTC [39].

Subsequently, various research teams detected additional *RET* rearrangements across multiple types of solid tumors [40].

Errors occurring during the repair of double-stranded DNA breaks are believed to be the source of *RET* fusion or rearrangement. These aberrations arise from mechanisms such as break-induced replication and non-homologous end-joining, which leads to chromosomal rearrangements or inversions that result in somatic *RET* gene fusions [14].

These fusions combine the RET intracellular kinase domain (located in the 3′ region) with the N-terminal portion of another gene (located in the 5′ region) that contains dimerization domains, such as coiled-coil motifs, LisH (Lis1 homology domain), or a SAM (sterile α motif domain). This fusion promotes ligand-independent dimerization, leading to constant RET kinase activation. The most common location for *RET* breakpoints is within intron 11, with less frequent occurrences in introns 7 and 10. So far, over 35 genes have been identified as *RET* fusion partners [2,14].

In PTC, *RET*/*PTC*1 (*CCDC6-RET*) and *RET*/*PTC*3 (*NCOA4-RET*) are the most frequently observed *RET* rearrangements, constituting over 90% of all identified gene rearrangements in *PTC* cases [25].

*CCDC6* (10q21.2) and *NCOA4* (10q11.22) are found on chromosome 10’s long arm, whereas *RET (10q11.21)* is transcribed in the opposite direction compared to *CCDC6* and *NCOA4*. Therefore, the formation of the *CCDC6-RET* and *NCOA4-RET* fusion is likely caused by a paracentric inversion on chromosome 10q [41].

*RET* rearrangements occur more frequently in PTCs induced by radiation. For example, 50–80% of PTC patients exposed to radioactive fallout from Chernobyl or the USA atomic bomb explosion in Japan have been shown to have these genetic changes. Compared to adults with PTC, children exhibit a higher incidence of these structural modifications [35].

In PTC cases, additional N-terminal partner genes include *PRKAR1A*, *GOLGA5*, *TRIM24*, *TRIM33*, *KTN1*, and *RFG9*. Among non-small cell lung cancer cases, *RET* fusion is found in 1–2% of patients, particularly those with adenocarcinoma histology. This genetic alteration is more prevalent in younger individuals who are typically diagnosed at or before age 60, and is often associated with little or no smoking history [42].

Among *RET* fusions observed in lung cancer, *KIF5B-RET* is the most prevalent, constituting roughly 80% of cases, while *CCDC6-RET* accounts for approximately 15%. Other less common fusion types include *NCOA4-RET*, *TRIM33-RET*, and *CUX1-RET* [20].

*KIF5B* is located on chromosome 10p11.22, suggesting that the formation of the *KIF5B-RET* fusion occurs through pericentric inversion of chromosome 10 [41].

- *RET* fusions result in the persistent activation of RET TK, enhancing cellular processes such as growth, viability, motility, and differentiation through the stimulation of various signaling pathways, including phosphoinositide 3-kinases (PI3K)/AKT, MAPK, and STAT3 [43].

*RET* fusion has been discovered in a wide array of cancer types through cutting-edge DNA and/or RNA sequencing methods. This genetic alteration has been identified not only in ovarian and salivary gland malignancies, but also in Spitz tumors, spitzoid melanomas, chronic myelomonocytic leukemia, and cancers of the colorectum and breast [35,36].

- Comprehensive studies examining colorectal and breast cancers have determined that *RET* fusion manifests in 0.2% (6/3117) [44] and 0.1% (8/9693) [45] of cases, respectively, based on extensive analytical data.

#### 2.3.4. *RET* Amplification

Other cytogenetic events, notably *RET* amplifications, have been observed in anaplastic thyroid cancer, PTC, and MTC; however, their significance in the development of thyroid carcinomas remains unclear [32].

A significant number of NSCLC patient tumor samples were investigated by the researchers, and a higher percentage of tumor samples exhibited low *RET* copy number gains (8.1%) and amplifications (2.8%) than *RET* rearrangements (0.7%). Amplifications were defined as either countless *RET* clusters or at least seven copies in more than 10% of cancer cells [46].

A comprehensive cancer study found that *RET* amplifications, characterized by at least six copies of wild-type *RET*, occurred in 0.16% (145/91,466) of analyzed tumor samples. Among NSCLC cases specifically, the prevalence was 0.13% (15/11,622). These amplifications have been documented in additional cancer types, such as hepatobiliary, prostate, and breast cancers [47].

The impact of these amplifications on elevated RET protein levels and their potential clinical relevance have not been thoroughly investigated [32].

## 3. RET in Cancers

### 3.1. Thyroid Cancer

Nearly 1.2% of individuals in the United States will receive a diagnosis of thyroid cancer at some stage in their life, and the anticipated number of cases globally in 2025 is projected to be around 300,000 [48,49,50,51]. A significant portion of PTCs occur as a result of chromosomal inversions or translocations, which activate *RET* (*RET*/*PTC* oncogenes), and approximately 50% with sporadic MTC carry somatic *RET* mutations. Thus, the RET proto-oncogene plays an important role in the pathogenesis, prognosis, and treatment of varying types of thyroid cancers.

#### 3.1.1. Medullary Thyroid Cancer

*RET* mutations are a key driver of MTC. Germline *RET* mutations are associated with hereditary variants of MTC such as MEN2A and MEN2B. On the other hand, Somatic *RET* mutations are found in approximately 50% of sporadic MTC cases [25,52,53].

#### 3.1.2. Hereditary MTC and RET Mutations (MEN2 Syndromes)

MEN2 is a group of inherited autosomal dominant cancer syndromes that significantly increase the risk of early-onset MTC. MEN2 syndromes are subclassified into MEN2A and MEN2B: both mutations in the *RET* gene. MEN2A, the most common subtype, accounts for approximately 95% of cases and includes FMTC, which was previously considered a separate subtype. MEN2A features MTC in all patients and may be associated with pheochromocytomas, parathyroid issues, cutaneous lichen amyloidosis, and Hirschsprung disease. In contrast, MEN2B is a more severe form with an earlier onset of MTC, making up roughly 5% of MEN2 cases [14]. MEN2B lacks parathyroid involvement; however, patients may present with marfanoid features, mucosal neuromas, and gastrointestinal manifestations [54].

Over 60 *RET* mutations have been identified, most being gain-of-function mutations in *RET* exons 5–16 leading to continuous RET kinase activity. About 95% of MEN2A cases are caused by substitutions in cysteine residues of the RET extracellular domain (C609, C611, C618, C620, C634), which are crucial for the structural stability and kinase function through disulfide bridges. These mutations disrupt intramolecular disulfide bonds promoting receptor dimerization and subsequent kinase activation without the need for a ligand. The C634 mutation is responsible for approximately 85% of MEN2A cases [14,54].

In rarer MEN2A mutations, variants such as *G533C* in the *RET* extracellular domain (E768, L790, V804, S891) and mutations in the *RET* intracellular domain are linked to a delayed onset of MTC [8]. In MEN2B, around 95% of cases involve the M918T mutation in RET’s catalytic domain. This mutation causes the kinase activation loop to open, leading to faster autophosphorylation. FMTC can result from mutations in both the extracellular and intracellular domains, including cysteine substitutions in the CRD and kinase domain-activating mutations. FMTC mutations, especially at the gatekeeper valine (V804), make the kinase resistant to certain inhibitors [54]. Another mutation, *A883F*, found in MEN2B patients, is in the RET kinase domain and increases activation and signaling, though it results in a less aggressive phenotype than M918T. Additionally, rare dual mutations like *V804M* and *Y806C* can enhance RET activity, but these are also associated with a less severe phenotype compared to *M918T* [14].

#### 3.1.3. Somatic *RET* Mutations in Sporadic MTC

Somatic *RET* mutations are present in about 40–50% of sporadic MTC cases and are linked to more aggressive disease and poorer prognosis [55]. The most common mutation is *M918T* in exon 16, which is associated with a worse clinical outcome, although mutations in other exons like 10, 11, 13, 14, and 15 are also seen, but less frequently [55,56,57,58]. These mutations are correlated with larger tumor sizes, advanced disease stages, and a higher likelihood of lymph node and distant metastases [55,58]. Specifically, the *M918T* mutation is particularly linked to an increased risk of lymph node metastasis and persistent disease. Patients with somatic *RET* mutations, especially *M918T*, tend to have a worse prognosis, including lower survival rates and higher recurrence rates, and these mutations are considered an independent factor for poor outcomes [55]. The discovery of somatic *RET* mutations has significant therapeutic implications, as targeted treatments like selective RET inhibitors, such as Selpercatinib, have proven effective in treating advanced MTC with *RET* mutations [59].

#### 3.1.4. Papillary Thyroid Cancer

PTC, the most common of thyroid carcinomas, accounts for nearly 80% of all cases [60]. Mutations in *RET* itself are less common in *PTC*; however, rearrangements of the gene, leading to *RET* fusions with other genes, are frequently observed [61]. These rearrangements result in the creation of constitutively active RET kinases, which leads to the abnormal activation of downstream signaling pathways, including the MAPK/ERK pathway, which is essential for cell proliferation. The fusion proteins resulting from these rearrangements lack the transmembrane domain, which is typically present in the normal RET protein, and instead localize to the cytosol, where they continuously activate RET kinase. *RET*/*PTC* rearrangements induce ligand-independent dimerization of the RET receptor, triggering the MAPK signaling cascade. This cascade involves the phosphorylation of molecules like RAS, RAF, and MEK, leading to the activation of ERK. The subsequent phosphorylation of ERK alters gene expression to promote cell proliferation, survival, and transformation. *BRAF*, a critical kinase in this pathway, is often co-activated in *RET*/*PTC*-associated PTC, amplifying the MAPK pathway’s activity [62,63,64].

Additionally, *RET*/*PTC* rearrangements can activate the PI3K/Akt pathway, which plays a crucial role in cell survival, growth, and metabolism. This activation enhances resistance to apoptosis and supports tumorigenesis by inhibiting pro-apoptotic factors while promoting cell survival signals. The intersection of this pathway with other signaling cascades adds complexity to the progression of thyroid cancer.

Another critical aspect of *RET*/*PTC* rearrangements is the ligand-independent activation of the RET receptor. In its wild-type form, RET requires ligand binding for activation. However, in *RET*/*PTC* rearrangements, fusion with various partner genes results in the loss of the extracellular ligand-binding and juxtamembrane domains, leading to constitutive activation of RET. This altered RET protein promotes uncontrolled signaling through its intracellular kinase domain, activating downstream networks like MAPK and PI3K/Akt, which further drive unregulated cell growth [3].

Moreover, *RET*/*PTC* rearrangements often involve gene fusions with proteins such as coiled-coil domain containing gene 6 (*CCDC6*), forming the *RET*/*PTC*1 RET fusion, and nuclear receptor co-activator gene 4 (*NCOA4*), forming the *RET*/*PTC*3 RET fusion. These fusion proteins not only facilitate ligand-independent signaling but can also disrupt the function of the fused genes. For instance, *NCOA4*, a fusion partner in certain *RET*/*PTC* variants, is involved in transcriptional regulation and affects thyroid cell differentiation and survival by interacting with nuclear hormone receptors [60]. *RET*/*PTC* rearrangements are also associated with pro-inflammatory signaling, which is a hallmark of *PTC*. These rearrangements lead to the upregulation of various cytokines such as IL-6, thereby facilitating tumor progression and metastasis [65]. Among the various types of *RET* fusions, *RET*/*PTC*1 and *RET*/*PTC*3 are the most prevalent, accounting for most rearrangements in *PTC*. The oncogenic nature of these fusions is well-documented, with transgenic models showing that the overexpression of RET/*PTC*1 can lead to the development of thyroid carcinomas that are histologically similar to human *PTC* tumors [15]. Furthermore, some RET fusion partners, such as *PRKAR1A* in RET/*PTC*2, are tumor suppressors. Fusion with RET leads to the loss of their tumor-suppressive function, contributing to thyroid tumorigenesis. *PRKAR1A*, which regulates PKA activity, is crucial for maintaining normal cellular functions. Its loss promotes the activation of pathways favoring cell proliferation and survival, further driving the oncogenic process [66].

The frequency of RET/*PTC* rearrangements in *PTC* varies globally, with studies reporting rates from 3% to as high as 85%, depending on geographic region and patient demographics. These alterations are more common in certain *PTC* subtypes, especially the classic and follicular variants, and are frequently found in tumors that are smaller than 1 cm in diameter, known as microcarcinomas. Moreover, *RET*/*PTC* rearrangements are rare in benign thyroid tumors and other types of thyroid carcinoma, such as follicular and medullary carcinoma. Notably, the prevalence of *RET*/*PTC* rearrangements is higher in pediatric PTC cases, particularly in populations exposed to radiation, such as those affected by the Chernobyl disaster. For example, while the most frequent genetic alteration in PTC is the *BRAF* V600E mutation, *RET*/*PTC*’s oncogenic effects require *BRAF*, and *RET*/*PTC*-associated tumors are typically more common in younger patients and present at earlier stages, while *BRAF* mutations are linked to older patients and more advanced tumors. This suggests a strong association between ionizing radiation and the development of *RET*/*PTC* fusions, with radiation-induced DNA damage promoting chromosomal rearrangements [60,67].

### 3.2. Non-Small Cell Lung Cancer

*RET* rearrangements are oncogenic drivers in 1–2% of non-small cell lung cancer (NSCLC) cases, primarily in the form of RET fusions, with the *KIF5B*-RET fusion accounting for 50–70% of RET-positive cases [68,69]. Other fusion partners, such as *CCDC6* and *NCOA4*, occur less frequently. As mentioned previously, these fusions lead to the activation of signaling pathways like MAPK, PI3K/AKT, and JAK/STAT, driving cell proliferation and tumor growth. RET fusions are primarily found in lung adenocarcinoma and are more prevalent among younger, non-smoking, female patients, particularly those of Asian descent. However, clinical patterns can vary across ethnicities, with some studies observing higher rates of RET fusions in male smokers, especially in Caucasian populations, indicating possible ethnicity-specific disease features [70].

RET fusion-positive NSCLC represents a distinct molecular and clinicopathological subtype when compared to other common oncogenic mutations, such as those in *EGFR* or *ALK*. Tumors with RET fusions are often poorly differentiated and have a higher likelihood of brain metastases. Research has shown that 27% of patients with RET fusion-positive NSCLC present with brain metastases at diagnosis, a figure that rises to 49% over the course of the disease. These findings highlight the necessity of monitoring for brain metastasis in patients with RET-rearranged adenocarcinomas [3,71].

Additionally, RET fusions can lead to resistance to targeted therapies, including Osimertinib, which emphasizes the need for personalized treatment approaches. As the identification of RET fusions becomes more widespread, the development of targeted therapies, particularly RET-specific TKIs, is emerging as a critical area of research to improve outcomes for RET fusion-positive NSCLC, including addressing complications like brain metastases [71].

### 3.3. Others

RET is altered and potentially actionable in other solid tumor types; however, its occurrence is much less frequent.

#### 3.3.1. RET in Breast Cancer

*RET* alterations occur in approximately 1.2% of breast cancer cases, with 66% being *RET* amplifications and 7% involving activating fusions. The most common fusions, *CCDC6-RET* and *NCOA4*-*RET*, promote tumor development by enhancing cell proliferation and survival [3]. RET, when activated by its ligand GDNF, can lead to estrogen-independent activation of ERα transcriptional activity, promoting cell survival even in the absence of estrogen. A study by Plaza-Menacho et al. indicated that targeting RET can sensitize ER+ breast cancer cells to tamoxifen, possibly overcoming endocrine resistance. The downregulation of RET significantly increased the sensitivity of MCF7 breast cancer cells to tamoxifen, while stimulation of RET by GDNF provided a protective effect against the drug [72]. In triple negative breast cancer (TNBC), RET is not directly considered a key factor in tumorigenesis; however, studies suggest that MKIs targeting RET can suppress tumor growth in TNBC models [3,73,74].

#### 3.3.2. RET in Prostate Cancer

Prostate cancer is the second leading cause of cancer-related deaths among men in the United States. While *RET* overexpression is commonly seen in prostate cancers, *RET* alterations account for only 1–2% of cases. Research has demonstrated that RET protein is overexpressed in high-grade prostatic intraepithelial neoplasia (PIN) and prostate cancer compared to benign tissue, and its expression correlates positively with the Gleason score, suggesting that RET overexpression plays a role in the malignant progression of prostate cancer. Elevated RET activity in prostate cancer may also be linked to increased secretion of GDNF by prostatic stromal cells or the release of GFRα1 by peripheral nerves, which can contribute to perineural invasion, a factor associated with poor prognosis [3]. In 2020, VanDeusen and colleagues found that aggressive, androgen-independent prostate cancers can transdifferentiate into neuroendocrine prostate cancers (NEPC), which show tumor-promoting RET activity that can be targeted with selective RET inhibitors [75]. These findings highlight the potential of targeting abnormal RET activity as a therapeutic strategy in prostate cancers, including those with neuroendocrine characteristics.

#### 3.3.3. RET in Colorectal Cancers

RET signaling plays a significant role in colorectal cancer (CRC), particularly *RET* gene fusions such as *NCOA4-RET* and *CCDC6*-*RET*, and *RET* mutations. RET fusions only constitute less than 1% of cases; however, they lead to constitutive activation of the RET pathway, promoting oncogenic signaling and tumor progression. Furthermore, RET fusion-positive CRCs are often characterized by right-sided tumors, microsatellite instability-high (MSI-RET) status, and the absence of concurrent *RAS* and *BRAF* mutations [44,75,76]. Despite research supporting an oncogenic role for RET alterations in CRC, its role has remained controversial. For example, RET mutations have been found to be potential tumor suppressors in CRC. In 2013, Luo et al. reported enhanced methylation and consequent downregulation of *RET* in CRC samples, suggesting a tumor-suppressing effect [77]. This was further supported by a 2021 study demonstrating reduced *RET* expression in CRC tissue compared to adjacent normal tissue [78]. Thus, RET signaling in colorectal cancer involves both oncogenic *RET* fusions and tumor-suppressive *RET* mutations.

#### 3.3.4. RET in Pancreatic and Ovarian Cancers

*RET* alterations occur in approximately 1.9% of pancreatic cancers whereas wild-type *RET*, co-receptor GFRα1, and GDNF are overexpressed in 50–70% of pancreatic cancer cases. Research also suggests that RET is linked to tumor aggressiveness and perineural invasion. While RET alterations are rare in pancreatic cancers, given that first RET fusion-positive pancreatic cancer patient was reported in 2021, patients with aberrant RET pathway activity may benefit from RET-targeted therapies [3,79].

Additionally, *RET* is altered in nearly 1.2% of ovarian cancers and has been identified as an oncogenic driver via *RET* missense mutations, such as R693H and A750T, which drive RET signaling. These mutations activate the MAPK and AKT pathways promoting tumorigenesis. RET inhibitors, such as vandetanib, have been shown to reduce RET-MAPK signaling, further highlighting it as a potential therapy for epithelial ovarian cancer [3,80].

## 4. Targeted Therapies

### 4.1. Mechanism of Action

As the landscape of oncology continues to evolve, specifically in regard to aberrant RET kinases activation in solid tumors, the treatment landscape has also evolved. MKIs were the first class to show potential in targeted treatment with moderate selectivity to RET. Although initially designed to target other kinases, these were repurposed due to their inhibitory actions on RET [6].

### 4.2. Multi-Kinase Inhibitors with Non-Selective RET Activity

#### 4.2.1. Cabozantinib

Cabozantinib is an oral MKI with potent activity against vascular endothelial growth factor (VEGF) receptor 2, as well as a number of other RTKs, including RET [81]. Cabozantinib has been shown to inhibit critical processes involved in angiogenesis and tumorigenesis, including tubule formation, cellular migration, and invasion [81]. In a phase II single-arm trial in the USA, Drilon et al. evaluated Cabozantinib in patients with *RET*-rearranged non-small cell lung cancer. Partial responses were observed in 7 out of 25 patients, resulting in an ORR of 28% and PFS of 5.5 months [82]. Cabozantinib was first FDA approved in 2012 for progressive metastatic MTC, based on the XL184-001 and EXAM trials, which demonstrated an acceptable safety profile and superior progression-free survival in the RET mutated subgroup [83]. Exploratory analysis revealed even greater benefit in patients with RET *M918T*-positive disease, with an OS of 44.3 months compared to 18.9 months in non-mutated patients [84]. Cabozantinib is also FDA approved for renal carcinoma and hepatocellular carcinoma. In summary, Cabozantinib has shown efficacy in *RET*-rearranged NSCLC and *RET*-mutant MTC.

#### 4.2.2. Vandetanib

Vandetanib is an oral MKI that targets TKs including EGFR-, VEGF- and RET-dependent signaling [85]. Initially, it was shown to inhibit VEGF signaling and angiogenesis, thereby reducing tumor cell growth [86]. Subsequent studies demonstrated its potent inhibition of RET-related signaling pathways, including *RET/PTC*, *RET/MEN2A*, and *RET/EGFR* in transformed NIH3T3 cells, leading to reduced proliferation and morphological reversion [87]. Vandetanib also targets various *RET* mutations, making it effective in treatment of patients with medullary thyroid carcinoma associated with MEN2A/2B, FMTC, and PTC [88]. It is FDA approved in metastatic or unresectable locally advanced medullary thyroid cancer, based on the ZETA trial, which demonstrated superior PFS for 30.5 months compared to 18.3 months with placebo [89]. However, follow-up studies have identified certain mutations which confer resistance to Vandetanib, which will be discussed in more detail later.

#### 4.2.3. Lenvatinib

Lenvatinib is an oral multi-targeted TKI that targets VEGF receptors 1–3, fibroblast growth factor (FGF) receptors 1–4, platelet-derived growth factor receptor alpha (PDGFRa), KIT, and RET. Preclinical studies demonstrated that Lenvatinib inhibited the phosphorylation of the RET fusion proteins and suppresses the growth of *RET* fusion-driven tumor models [90]. In a phase II trial, 25 out of 536 patients with *RET* translocations were treated with Lenvatinib and yielded an ORR of 16%. Despite the modest response rate, treatment with Lenvatinib improved the median PFS in these patients [91]. Lenvatinib received FDA approval in patients with iodine-131-refractory thyroid cancer in 2015 [3], followed by approvals in 2018 for advanced or metastatic hepatocellular carcinoma [92]. It was later approved for the treatment of advanced renal cell carcinoma in combination with Everolimus [93] and for advanced endometrial cancer in combination with Pembrolizumab [94]. In summary, while Lenvatinib has shown activity in RET fusion-positive cancers, its response rate is relatively modest compared to more selective RET inhibitors. However, it remains a valuable treatment option for certain *RET*-mutated cancers, particularly in the context of thyroid cancer.

#### 4.2.4. Sorafenib

Sorafenib is an oral MKI that was first shown to inhibit intracellular Raf kinases and other kinase receptors including VEGF, PDGFR-beta, and cKIT [95,96]. Subsequent studies demonstrated that Sorafenib inhibits RET kinase functioning and signaling of both wild type and oncogenic RET in MEN2 tumor cells. It promotes RET lysosomal degradation independently of proteasomal targeting and inhibits RET/*PTC* rearrangements in PTC cells [97,98]. Sorafenib received FDA approval in 2005 for treatment of advanced renal cell carcinoma, followed by approval for the treatment of unresectable hepatocellular carcinoma [99,100]. In a phase II trial, evaluating Sorafenib in MTC, the partial response rate was 6.3%, with a median progression free survival (PFS) of 17.9 months. Sorafenib was evaluated in the treatment of RET fusion-positive NSCLC; patients received 400 mg of Sorafenib twice daily, but the resulting responses were limited and demonstrated minimal clinical benefit in RET fusion-positive NSCLC patients [101]. In summary, while Sorafenib shows some activity in *RET*-mutated cancers, its efficacy in RET fusion-positive NSCLC remains limited and further testing is required.

#### 4.2.5. Regorafenib

Regorafenib is an oral MKI that targets VEGF, TIE2, PDGFR-beta, KIT and RET, which play key roles in tumor angiogenesis, oncogenesis and tumor microenvironment. Neuroblastomas (NB), which frequently overexpress RET, are associated with poorer outcomes when RET expression is elevated [102]. Chen et al. demonstrated that Regorafenib inhibits the RET-mediated PI3K/protein kinase B (AKT)/mechanistic target of rapamycin (mTOR) signaling pathway in NB cells [102]. Additionally, it targets the RET-Src axis, inhibiting the JAK1/2-STAT1 and MAPK signaling pathways and simultaneously reducing expression of PD-L1, potentially enhancing the immune response to tumor cells [103]. While Regorafenib has shown antitumor activity in *RET*-mutated cancers, particularly neuroblastoma, its clinical benefit remains limited, necessitating further clinical investigation. Regorafenib is Food and Drug Administration (FDA) approved in metastatic colorectal cancer, gastrointestinal stromal tumors and hepatocellular carcinoma (HCC).

#### 4.2.6. Sunitinib

Sunitinib is an oral MKI that inhibits VEGFR, PDGFR, KIT, FLT3 and RET, exhibiting both antitumor and antiangiogenic properties. PTC cells harboring *RET*/*PTC* rearrangement demonstrated the ability to inhibit tumor growth through the suppression of an upstream MAPK signaling cascade [104]. Additionally, data from a global registry of patients with *RET*-rearranged NSCLC reported a complete or partial response of 22%, indicating limited activity in comparison to more selective RET inhibitors [105]. Despite the limited clinical benefit in *RET* mutated cancers, it is FDA approved in the treatment of GI stromal tumors, advanced pancreatic neuroendocrine tumors and renal cell carcinoma.

#### 4.2.7. Alectinib

Alectinib is an oral ATP-competitive TKI that targets anaplastic lymphoma kinase [1] and can inhibit the ALK gatekeeper mutation *L1196M* [106]. Additionally, alectinib demonstrated activity against the RET wild-type, suppressing phospho-RET showing antitumor activity in mouse models of *RET*-fusion driven tumors, including *RET*-rearranged NSCLC [107]. However, clinical studies have shown limited efficacy in *RET*-rearranged NSCLC [108]. In a study by Takeuchi et al., alectinib achieved an objective response of only 4% and progression-free survival of 3.4 months [109]. Similarly, the ETOP ALERT-lung trial reported no objective responses among 14 patients, with a median PFS of 3.7 months and a disease stabilization rate of 23% for 24 weeks. The trial was terminated early due to discouraging results and the emergence of more potent selective RET inhibitors [110]. While alectinib received accelerated FDA approval in 2015 for treatment of patients with ALK-positive NSCLC who progressed on crizotinib, its role in RET-rearranged NSCLC remains limited due to the availability of more selected RET inhibitors [1].

### 4.3. Selective RET Inhibitors

While the previously described MKIs showed some selective activity targeting RET, the clinical benefits remained suboptimal in patients with *RET* mutations. In contrast, selective RET inhibitors have been specifically designed to target aberrant *RET* mutations while minimizing off-target effects associated with other MKIs. As described below, this emerging class of inhibitors has shown significant clinical benefits.

#### 4.3.1. Selpercatinib

Selpercatinib is selective kinase inhibitor that targets wild-type RET and multiple mutated RET isoforms [111]. It inhibits *RET* fusion oncogenes by preventing homodimer formation and the autophosphorylation of specific TKs, thereby disrupting key signaling pathways involved in cellular survival, proliferation and growth [112]. A preclinical study demonstrated potent and selective anti-RET activity in human cancer cells with *RET* gene alterations, with clinical responses observed in patients with metastatic medullary thyroid cancer and metastatic non-small cell lung cancer [113]. LIBRETTO-001, an ongoing phase I/II clinical trial, evaluated selpercatinib in patients with RET alterations. In *RET* fusion-positive NSCLC, overall response rate (ORR) was 84% in treatment-naïve patients and 61% in those pretreated with platinum-based chemotherapy [114]. Notably, among 26 patients with measurable CNS metastases, the intracranial ORR was 85%, highlighting selpercatinib’s ability to penetrate the blood–brain barrier—an essential feature for NSCLC treatment [113]. For patients with *RET*-mutated medullary thyroid cancer with or without previous Vandetanib or cabozantinib treatment, the objective response was 69–79% [115]. Additionally, patients with NSCLC and MTC treated with selpercatinib reported a stable or improved quality of life [116,117]. In 2020, selpercatinib received FDA accelerated approval for patients with metastatic *RET* fusion-positive NSCLC, advanced or metastatic *RET*-mutant medullary thyroid cancer and advanced or metastatic *RET* fusion-positive thyroid cancer who require systemic therapy and who are radioactive iodine-refractory [111]. Most recently, results from the phase III LIBRETTO-431 trial demonstrated that selpercatinib significantly prolonged PFS compared with platinum-based chemotherapy ± pembrolizumab in treatment-naïve *RET* fusion-positive NSCLC, establishing selpercatinib as the new standard first-line therapy for this molecularly defined population. Multiple ongoing trials are further evaluating selpercatinib’s efficacy in *RET*-mutated cancer.

#### 4.3.2. Pralsetinib

Pralsetinib is a highly selective RET inhibitor that targets wild-type as well as kinase-activating RET mutations and fusions, which act as oncogenic drivers promoting tumor cell proliferation [118]. The ARROW clinical trial, initiated in 2017, is a phase I/II study conducted at 71 sites evaluating patients with locally advanced or metastatic solid tumors, including *RET* fusion-positive NSCLC [119]. Among 281 patients with *RET* fusion-positive NSCLC, the ORR was 72% in treatment-naïve patients and 59% in those previously treated with platinum-based chemotherapy [119]. Notably, pralsetinib demonstrated the ability to cross the blood–brain barrier and the response, achieving an intracranial response rate of 70% in those with measurable CNS metastases [120]. In patient with *RET*-mutant medullary thyroid cancer, the ARROW trial reported ORR of 60% in those previously treated with cabozantinib or vandetanib, and 71% in treatment-naïve patients. The long-term follow-up of ARROW confirmed durable responses and manageable toxicity across *RET*-altered thyroid cancers, with hypertension, neutropenia, and anemia being the most common grade ≥3 treatment-related adverse events, and a low rate of discontinuation due to adverse events (<5%) [121]. In 2020, pralsetinib received FDA accelerated approval for metastatic *RET* fusion-positive non-small cell lung cancer (NSCLC) and for advanced or metastatic *RET*-mutant medullary thyroid cancer [118]. Ongoing trials continue to evaluate pralsetinib’s efficacy in *RET*-mutated cancer.

## 5. Toxicities and Side Effects of Targeted Therapy

While RET-targeted therapy has become a cornerstone in the treatment of RET altered malignancies, these therapies are not without a toxicity profile and adverse effects.

As mentioned previously, multi-kinase inhibitors such as vandetanib, cabozantinib, lenvatinib, and sorafenib target RET, alongside other kinases like VEGFR, EGFR, Mesenchymal–Epithelial Transition factor (MET), KIT Proto-Oncogene (KIT), and AXL Receptor Tyrosine Kinase (AXL), which leads to a broader range of adverse effects than selective RET inhibitors [122].

### 5.1. Cardiovascular Side Effects

Multi-kinase RET inhibitors exhibit several cardiovascular side effects. Vandetanib, cabozantinib, lenvatinib, regorafenib, and sunitinib have all been associated with QT interval prolongation, which can lead to torsade de pointes and sudden cardiac death. These RET inhibitors, in addition to alectinib, are also associated with hypertension [123,124,125]. Furthermore, Lenvatinib is associated with cardiac failure and acute coronary syndrome. Sorafenib, on the other hand, is associated with acute myocardial infarction, acute coronary syndrome, atrial fibrillation, and aortic dissections [126]. Sunitinib increases the risk of left ventricular dysfunction [126]. Alectinib, although primarily an ALK inhibitor, can cause bradycardia [123,124]. Thus, patients on these agents require close monitoring of blood pressure and regular electrocardiograms, as recommended by the American Society of Clinical Oncology [127].

### 5.2. Gastrointestinal Side Effects

RET inhibitors also demonstrate an array of gastrointestinal side effects. These include diarrhea, nausea, vomiting, anorexia, and decreased appetite [85,128,129,130,131,132,133,134]. Vandetanib and aletinib can cause constipation as well, while lenvatinib, sorafenib, Regorafinib, and sunitinib have been associated with stomatitis [130,132,133]. As a result, the NCCN guidelines emphasize the importance of follow-up and monitoring of these side effects in patients on these agents [135].

### 5.3. Endocrine and Metabolic Side Effects

Endocrinologic and metabolic side effects have also been associated with RET inhibitor therapy. Hypothyroidism, hyperglycemia, and dyslipidemia commonly occur because of nearly all RET inhibitor therapy, whether it be a multi-kinase inhibitor or selective RET inhibitor [35,136,137,138,139,140]. Despite this, selective RET inhibitors such as selpercatinib and pralsetinib are generally more tolerated [139,140]. The European Society of Endocrinology recommend careful monitoring of thyroid hormone, glucose, and lipid levels [136].

### 5.4. Renal and Hepatic Side Effects

Nearly all RET inhibitor agents are associated with elevated transaminase and creatinine levels as well as proteinuria, necessitating careful monitoring as per the American Society of Clinical Oncology. Sorafenib and regorafenib, specifically, are associated with severe liver injury [139,140,141,142,143,144,145,146].

### 5.5. Psychiatric Side Effects

RET inhibitor therapy has also been associated with psychiatric side effects, most commonly anxiety, depression, and insomnia [139,147,148,149]. Vandetanib, although a MKI, has a relatively lower incidence of psychiatric side effects when compared to other MKIs [147]. Sorafenib, on the other hand, has been associated with cognitive impairment and psychiatric side effects are commonly severe enough to require dose adjustments or therapy discontinuation. Sunitinib’s psychiatric side effects have also been severe enough to lead to hospitalization and even death in some cases. Regular mental health evaluation is necessary in patients on these agents, as recommended by the American Society of Clinical Oncology: particularly with sorafenib, sunitinib, and cabozantinib [147,148].

### 5.6. Hematology Side Effects

Myelosuppression and subsequent anemia, neutropenia and thrombocytopenia are unfortunately common side effects of RET inhibitor therapy, particularly sunitinib. Furthermore, RET inhibitor therapy is associated with secondary malignancies such as squamous cell carcinoma, necessitating regular dermatologic evaluations [35,135,143,150].

### 5.7. Other Side Effects

Other side effects of RET inhibitor therapy include fatigue and dermatologic side effects. Fatigue in this case is multifactorial. For example, sorafenib and sunitinib influence mitochondrial function, leading to decreased ATP production [151]. This, coupled with myelosuppression and endocrine side effects such as hypothyroidism, as discussed previously, can precipitate fatigue [15,16,17,18,34]. Rashes and hand–foot syndrome are common dermatologic side effects as well, especially with cabozantinib [152]. Severe skin reactions such as toxic epidermal necrolysis and Stevens–Johnson Syndrome have been reported with vandetanib [85]. Additionally, vandetanib and lenvatinib have been associated with reversible posterior leukoencephalopathy syndrome [153,154].

## 6. Resistance Mechanisms

While RET-targeted therapies have been effective, resistance has inevitably emerged through diverse mechanisms. This presents a significant challenge in the treatment of RET-altered cancers [155] (Table 1).

### 6.1. Primary and Acquired Resistance Mechanisms

Generally, resistance to RET inhibitor therapy can be primary or acquired. Primary resistance is intrinsic and occurs because of pre-existing genomic alterations that prevent drug effectiveness [156]. On the other hand, acquired resistance occurs after an initial period of response to RET inhibitor therapy. Acquired resistance is driven by multiple mechanisms. One mechanism is by acquired mutations known as *RET* solvent front mutations, which hinder the binding of selective RET inhibitors. For example, *RET G810* substitutions of the *RET* kinase domain specifically affect the efficacy of selpercatinib and pralsetinib by altering the ATP-binding pocket [157,158,159]. Furthermore, gatekeeper mutations such as *RET V804M* and *RET V804L* are also important mechanisms of resistance. These mutations hinder the binding of certain RET inhibitors at the ATP-binding site, thereby reducing their efficacy [160,161]. As a result, newer selective RET inhibitors, such as selpercatinib and pralsetinib, were designed to overcome these challenges. They exhibit a unique binding mode that prevents gatekeeper mutation interference, enabling them to maintain their efficacy. Despite this, resistance can still occur through other mechanisms [157,158,160,161,162]. *RET* solvent front mutations and gatekeeper mutations are classified as on-target mutations, given that they directly affect targeted drug binding.

Off-target resistance mechanisms occur through the activation of bypass signaling pathways such as MET amplification, EGFR pathway activation, and *KRAS* or *NRAS* mutations. MET amplification activates signaling pathways including MAPK and PI3K/AKT, driving tumor growth despite RET inhibition. Research has demonstrated that MET amplification can occur without RET resistance mutations, underscoring this as an independent resistance mechanism [156,157,160,163]. Furthermore, EGFR activation promotes resistance to RET inhibitors by way of ERK and AKT pathway activation. This has been demonstrated in CCDC6-*RET* lung cancer cells, where stimulation by EGF leads to resistance against sunitinb, vandetanib, and sorafenib. The use of EGFR inhibitors such as gefitinib, however, can restore sensitivity to RET inhibitors [157,164].

*KRAS* mutations similarly lead to reactivation of downstream signaling pathways such as MAPK, which can bypass the inhibitory effects of RET inhibitors. For example, one study on *RET* fusion-positive NSCLC patients treated with the selective RET inhibitors, selpercatinib and pralsetinib, found acquired *KRAS* amplification as a mechanism of resistance, allowing continued cell proliferation and survival. Moreover, resistance to the multi-kinase inhibitor ponatinib was mediated by an oncogenic *NRAS* mutation that led to reactivation of the MAPK pathway, driving cell proliferation [157,165].

### 6.2. Histological and Phenotypic Transformation

Histological and phenotypic transformation involves a change in the histological subtype or phenotypic characteristics of the tumor as a mechanism of resistance to RET inhibitor therapy, rendering RET inhibitors less effective. One example is the transformation of NSCLC to SCLC, though it is more commonly associated with resistance to EGFR inhibitors. Transformation to a squamous cell carcinoma subtype is another resistance mechanism, given that the molecular drivers and therapeutic targets of squamous cell carcinoma differ from those of adenocarcinoma. Phenotypic changes, including the epithelial to mesenchymal transition, are an additional resistance mechanism characterized by the increased resistance to targeted therapies, including RET inhibitors [156,157,166].

### 6.3. Therapeutic Strategies to Overcome Resistance

Several therapeutic strategies have been developed to overcome resistance to RET inhibitor therapy. Biomarker-based patient selection is essential for the effective use of RET inhibitors across cancer types. Comprehensive molecular profiling, preferably using next-generation sequencing (NGS) that interrogates both DNA and RNA, is recommended to identify *RET* alterations and guide therapy selection. RNA-based NGS offers greater sensitivity for detecting fusions, and broad genomic profiling is critical, given the heterogeneity of *RET* fusion partners and the tumor-agnostic efficacy of selective RET inhibitors [32]. Despite these advances, acquired resistance remains a major clinical challenge. Secondary *RET* mutations, particularly solvent front mutations (*G810C/S/R*) and gatekeeper mutations (*V804M/L*) and activation of bypass signaling pathways such as MET, EGFR, FGFR, and Hedgehog-Gli, can diminish inhibitor efficacy [32,167,168,169]. Novel next-generation inhibitors, including APS013118, are being developed to overcome these resistance mechanisms and improve CNS penetration. Combination therapies are also being investigated to enhance efficacy and overcome resistance, such as selpercatinib plus crizotinib for MET amplification-driven resistance, RET inhibitors with EGFR or MET inhibitors for bypass activation, and arsenic trioxide plus pralsetinib to inhibit Hedgehog-Gli signaling and restore sensitivity [167]. In cases of histologic transformation, shifting to a specific chemotherapy regimen that is typically used for small cell lung cancer may be required [156,157,170]. Collectively, the integration of biomarker-driven patient selection, next-generation RET inhibitors, and rational combination strategies is transforming the precision management of *RET*-altered malignancies.

**Figure 2:**
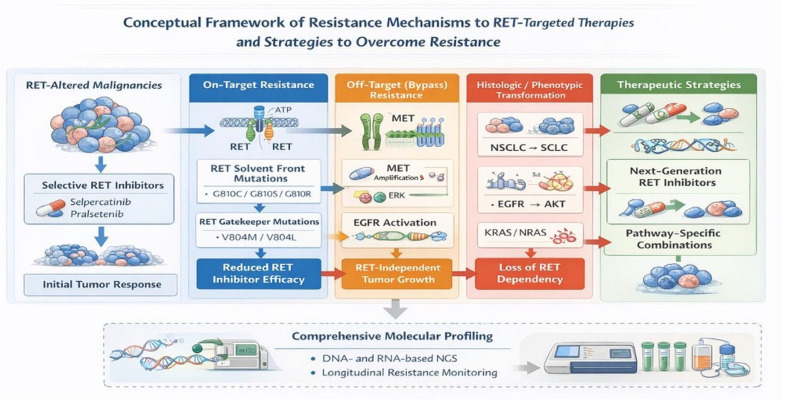
RET-altered malignancies initially respond to selective RET inhibitors such as selpercatinib and pralsetinib; however, resistance frequently develops through multiple mechanisms. On-target resistance arises from secondary *RET* mutations, including solvent-front mutations (e.g., *RET* G810 substitutions) and gatekeeper mutations (e.g., *RET* V804M/L), which reduce inhibitor binding and efficacy. Off-target resistance occurs through activation of bypass signaling pathways, such as MET amplification, EGFR pathway activation, or mutations in downstream effectors including *KRAS* and *NRAS*, enabling continued tumor growth despite RET inhibition. Histologic and phenotypic transformation, including transformation from non–small cell lung cancer (NSCLC) to small cell lung cancer (SCLC) or epithelial-to-mesenchymal transition, may also contribute to therapeutic resistance. Emerging strategies to overcome resistance include the development of next-generation RET inhibitors and rational combination therapies targeting bypass signaling pathways. Comprehensive molecular profiling using DNA- and RNA-based next-generation sequencing (NGS) is critical for identifying resistance mechanisms and guiding precision treatment strategies.

Next-generation RET inhibitors, for example, are designed to overcome specific resistance mutations, including the solvent front mutations discussed previously. Selective RET inhibitors such as selpercatinib and pralsetinib have shown efficacy against gatekeeper mutations like *RET V804M/L*. Regarding resistance mechanisms involving bypass signaling pathways, combination therapies are being explored. For example, combining RET inhibitors with EGFR inhibitors can overcome the resistance mediated by EGFR activation. Similarly, combining RET inhibitors with MET inhibitors can overcome resistance due to MET amplification. Resistance because of histological transformation can be overcome by a change in therapeutic strategy to include specific chemotherapy regimens that are typically used for SCLC [156,157,167] (Figure 2).

## 7. Conclusions

In conclusion, selective RET inhibitors have transformed the management of RET-altered cancers, yet primary and acquired resistance remain major challenges. Ongoing efforts to develop next-generation inhibitors, optimize biomarker-driven patient selection, and expand data across rare tumor types will be key to maximizing the clinical impact of RET-targeted therapy.

## Figures and Tables

**Table 1:** Comparison of multi-kinase and selective RET inhibitors, including FDA approval year, key efficacy outcomes, and representative adverse events.

Drug	Class	Targets	FDA	Key Efficacy	Common AE
Cabozantinib	Multi-kinase	RET, VEGFR2	2012	ORR 28% (RET-NSCLC)	QT prolongation, HTN, GI
Vandetanib	Multi-kinase	RET, EGFR, VEGFR	2011	MTC: ↑ PFS	QT prolongation, HTN, Skin
Lenvatinib	Multi-kinase	RET, VEGFR, FGFR	2015	ORR 16%	HTN, Cardiac
Sorafenib	Multi-kinase	RAF, VEGFR	2005	Limited RET benefit	CV, GI
Sunitinib	Multi-kinase	RET, VEGFR	2006	ORR ~22%	QT prolongation, Cytopenia
Selpercatinib	Selective RET	RET only	2020	ORR 84%(TN)	GI, LFTs
Pralsetinib	Selective RET	RET only	2020	ORR 72% (TN)	HTN, Neutropenia

## Data Availability

No new data were generated or analyzed in this study.
